# Supersonically Spray-Coated Colloidal Quantum Dot Ink Solar Cells

**DOI:** 10.1038/s41598-017-00669-9

**Published:** 2017-04-04

**Authors:** Hyekyoung Choi, Jong-Gun Lee, Xuan Dung Mai, Matthew C. Beard, Sam S. Yoon, Sohee Jeong

**Affiliations:** 1 Nano-Mechanical Systems Research Division, Korea institute of Machinery and Materials (KIMM), Daejeon, 34103 Korea; 2 Korea University of Science and Technology (UST), Daejeon, 34113 Korea; 3 School of Mechanical Engineering, Korea University, Seoul, 02841 Korea; 4 National Renewable Energy Laboratory, Golden, Colorado 80401 United States

## Abstract

Controlling the thickness of quantum dot (QD) films is difficult using existing film formation techniques, which employ pre-ligand-exchanged PbS QD inks, because of several issues: 1) poor colloidal stability, 2) use of high-boiling-point solvents for QD dispersion, and 3) limitations associated with one-step deposition. Herein, we suggest a new protocol for QD film deposition using electrical double-layered PbS QD inks, prepared by solution-phase ligand exchange using methyl ammonium lead iodide (MAPbI_3_). The films are deposited by the supersonic spraying technique, which facilitates the rapid evaporation of the solvent and the subsequent deposition of the PbS QD ink without requiring a post-deposition annealing treatment for solvent removal. The film thickness could be readily controlled by varying the number of spraying sweeps made across the substrate. This spray deposition process yields high-quality n-type QD films quickly (within 1 min) while minimizing the amount of the PbS QD ink used to less than 5 mg for one device (300-nm-thick absorbing layer, 2.5 × 2.5 cm^2^). Further, the formation of an additional p-layer by treatment with mercaptopropionic acid allows for facile hole extraction from the QD films, resulting in a power conversion efficiency of 3.7% under 1.5 AM illumination.

## Introduction

Colloidal quantum dots (QDs) have shown great promise for use in optoelectronic applications such as photovoltaics and photodetectors owing to their size- and shape-tunable optical and electrical properties^1–3^. In addition, the high scalability of the devices based on solution processing as well as the fact that they can be integrated readily make this technique highly suited for commercial manufacturing. Lead chalcogenide (PbX, X = S, Se, and Te) QDs with tunable bandgaps spanning the visible to near-infrared (NIR) wavelength range are the most suitable for QD-based next-generation photovoltaics^4–6^.

Typically, QDs synthesized *via* colloidal routes are capped with long-chain organic ligands that act as an insulating layer; however, this prevents their direct use in electronic devices because of the weak interparticle coupling resulting from the insulating layer^7^. Thus, a post-synthesis ligand exchange is necessary for such QDs to find use in practical devices, wherein the initial bulky ligands are replaced with shorter ligands. Multistep sequential layer-by-layer (LBL) assembly, in which crack-free conductive films of desired thicknesses are formed using spin-coating, dip-coating, and spray-coating, is the most commonly used technique for fabricating devices such as solar cells and field-effect transistors (FETs)^8^.

Nevertheless, such LBL fabrication techniques for forming conductive films suffer from several associated issues. For instance, close-packed dense films that are free of cracks and voids formed because of the decrease in the volume of the ligands after the ligand exchange process are hard to produce^9–11^. Further, the spin-coating and dip-coating techniques are typically used; however, these only allow for small-scale batch processing and are incompatible with roll-to-roll manufacturing schemes. Recently, several groups have explored spray coating because it may potentially offer the advantages of a well-established industrial technique^12–14^. However, they used native-ligand-passivated QDs dispersed in organic solvents as the QD precursors, which required ligand exchange after deposition. In addition, while LBL assembly is, by far, the most suitable technique for forming films that exhibit the desired thickness, it generally involves significant losses of the QD solution and is a time-consuming process^13, 14^.

Recently, a strategy has been developed wherein the native ligands are replaced with shorter ligands in the solution phase itself; this strategy allows for the direct deposition of the QD solution onto the substrate, yielding conductive films without requiring a solid-state ligand exchange^9, 11, 15–19^. However, these QDs with short ligands, whose dispersions are termed as QD inks, do not exhibit colloidal stability, thus making it difficult to fabricate high-quality QD films by controlling certain parameters, such as the film thickness^15, 20^. Moreover, QD inks are typically dispersed in a high-boiling-point solvent such as propylene carbonate (PC) or dimethylformamide^16^. The use of such high-boiling-point solvents makes it difficult to fabricate QD films because removing the solvent during deposition is cumbersome. Therefore, a single-step-deposition process becomes inevitable; however, such processes do not allow for sophisticated control over the film thickness. Kim *et al*. recently fabricated QD solar cells using an iodide-terminated PbS QD ink as the light-absorbing layer. They used the spin-coating method to deposit the films and reported that the efficiency was limited by the thickness of the deposited layer, which was ~150 nm^15^.

In this study, we propose a new method for the deposition of conductive PbS QD films using PbS QD inks by a supersonic spray-coating method. The proposed method is a quick one as it involves rapid (supersonic) spraying. We used a PbS QD ink stabilized with MAPbI_3_, which are colloidally stable under ambient conditions, as determined through dynamic light scattering (DLS) measurements and absorbance spectra analysis. Using this deposition method, the film thickness can be tuned simply by varying the number of spraying passes made. Precursor wastage is minimized, because the precursor is deposited onto the substrate with high accuracy. Finally, the photovoltaic cells produced from the PbS QD ink by using this coating approach exhibit a power conversion efficiency (PCE) of 3.7%.

## Results and Discussion

### Synthesis of PbS QD ink

Oleate-capped PbS QDs were synthesized by using Pb(oleate)_2_ and bis(trimethylsilyl) sulfide^21^. PbS QDs with a diameter of 3 nm and exhibiting the 1^st^ excitonic transition peak at 880 nm (see absorbance spectra in Fig. S1) were used for fabricating solar cells. Before the ligand-exchange process, the PbS QDs were dispersed in octane, while the MAPbI_3_ precursor was dissolved in *N*-methylformamide (NMF).

**Figure 1:**
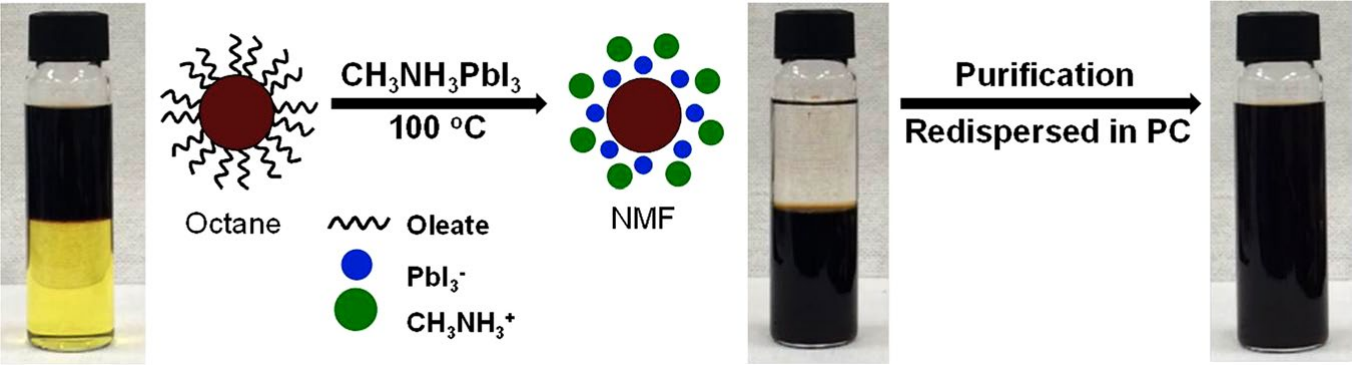
Illustration of phase transfer of PbS QDs from nonpolar solvent (octane) to polar solvent (NMF) induced by ligand exchange of oleates with CH_3_NH_3_PbI_3_ (MAPbI_3_). Acetone and PC were used for purification and redispersion, respectively.

To ensure fast and efficient ligand exchange in the concentrated PbS QDs-oleate solution (100 mg·mL^−1^, 10 mL), the QDs were added to a highly concentrated solution of the replacement ligand (1 M, 10 mL). The ligand-exchange process was accelerated by stirring at 100 °C (Fig. 1), in contrast to the conventional ligand-exchange process, which is performed at room temperature^11, 16^. NMF, which was used as the solvent for MAPbI_3_, has an ultrahigh dielectric constant (ε ≈ 182), which allows for electrostatic stabilization between the surfaces of the PbS QDs and the new MAPbI_3_ ligands. The complete phase transfer of the PbS QDs from octane to NMF took less than 10 min. To ensure compatibility between the two phases, acetone (ε ≈ 21) was used as the nonsolvent; as a result, the PbS QDs-MAPbI_3_ could be isolated by the centrifugation of the destabilized QD solution. PC was used as the solvent, in order to ensure the electrostatic stability of the concentrated PbS QDs-MAPbI_3_ solution (~100 mg·mL^−1^) after the removal of the free ligands by purification, because of its high dielectric constant (ε ≈ 64) as well as the weak desorption of the anions and the efficient solvation of the cations^16^. On the other hand, NMF was found to be unsuitable because it caused the strong desorption of the anions (PbI_3_), leading to a weak electrostatic double layer, even when the PbS QDs-MAPbI_3_ were dissolved in NMF before purification.

### Characterization of PbS QD ink in solution phase

**Figure 2:**
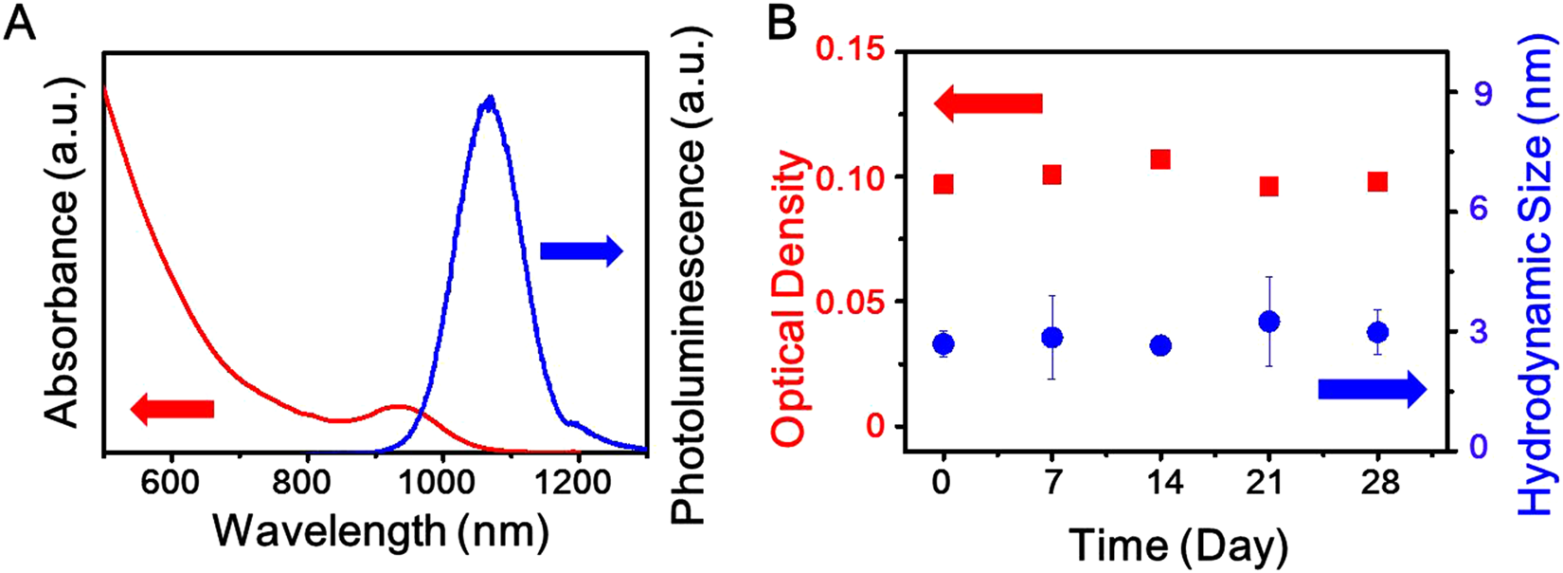
(**A**) Absorption (red line) and photoluminescence (blue line) spectra of PbS QDs-MAPbI_3_ dispersed in PC. (**B**) Changes in optical density corresponding to 1^st^ excitonic peaks (squares) as determined from absorption spectrum (Fig. S3) and hydrodynamic size (circles) as measured by DLS over time.

Figure 2A shows the absorption and emission spectra of the PbS QDs-MAPbI_3_ system. It can be seen that the absorption (~77 meV) and emission (~92 meV) spectra are slightly red-shifted compared to those of the PbS QDs-oleate (Fig. S1). Further, distinct absorbance features are observed after the ligand-exchange reaction at 100 °C, indicating that the size of the particles remains unchanged and that quantum confinement occurs without severe particle aggregation. Further, TEM images of the PbS QDs-MAPbI_3_ also confirm that the size of the particles is preserved after the ligand-exchange process and that the size distribution is narrow (Fig. S2). The colloidal and air stabilities of the QD ink were monitored based on the changes in the absorption peaks as well as the particle size over time as by determined by DLS measurements (Fig. 2B). DLS is a useful tool for monitoring the presence of aggregates and has been used widely for determining the hydrodynamic size of nanoparticles in colloidal suspensions^22^. The average hydrodynamic diameter of the particles in the PbS QD ink, as measured by DLS, was 3 nm, which is similar to the value obtained from the TEM measurements (Figs S2 and S3), with the particle size remaining constant over time (Fig. 2B, right). Furthermore, the optical density (OD) corresponding to the 1^st^ excitonic transition at 930 nm in the absorbance spectrum (Fig. S4) was monitored to determine whether particle aggregation occurred over time. The peaks remained constant and did not undergo broadening for more than four weeks, implying that the PbS QD ink exhibited high colloidal and air stabilities. In several previous studies, halide salts were used to improve the air stability of air-unstable lead chalcogenide QDs^23, 24^.

The ligand-exchange process was attempted using other precursors such as MAPbBr_3_, and MAPbCl_3_ dissolved in NMF. The results for the PbS QDs-MAPbBr_3_ system were similar to those obtained for the PbS QDs-MAPbI_3_ congener, as evidenced by the optical properties and TEM images (Figs S5 and S6). In contrast, the MAPbCl_3_ precursor induced particle aggregation, as determined by the results of absorbance, photoluminescence (PL), and TEM analyses (Figs S5 and S6). As per the concept of hard and soft acids and bases developed by Pearson, the soft acidic Pb atoms on the surfaces of the PbS QDs preferentially bind to the soft bases I and Br instead of the hard base Cl^25^.

The PbS QDs-MAPbI_3_ dispersed in PC were negatively charged, as indicated by the fact that their average ξ-potential was −13 mV, as determined by electrophoretic measurements (Fig. S7). Sayevich *et al*.^10^ reported that iodide-capped PbSe QDs formed from a NH_4_I precursor *via* a solution-phase ligand-exchange process have a ξ-potential of −28 mV. The QD ink-MAPbI_3_ particles were less negatively charged because an electrical double layer comprising PbI_3_ anions and MA cations is formed; this double layer encapsulates the QDs, suggesting that the surfaces of the synthesized QDs are well stabilized by the MAPbI_3_ ligands in the colloids.

### Fabrication of PbS QD films

**Figure 3:**
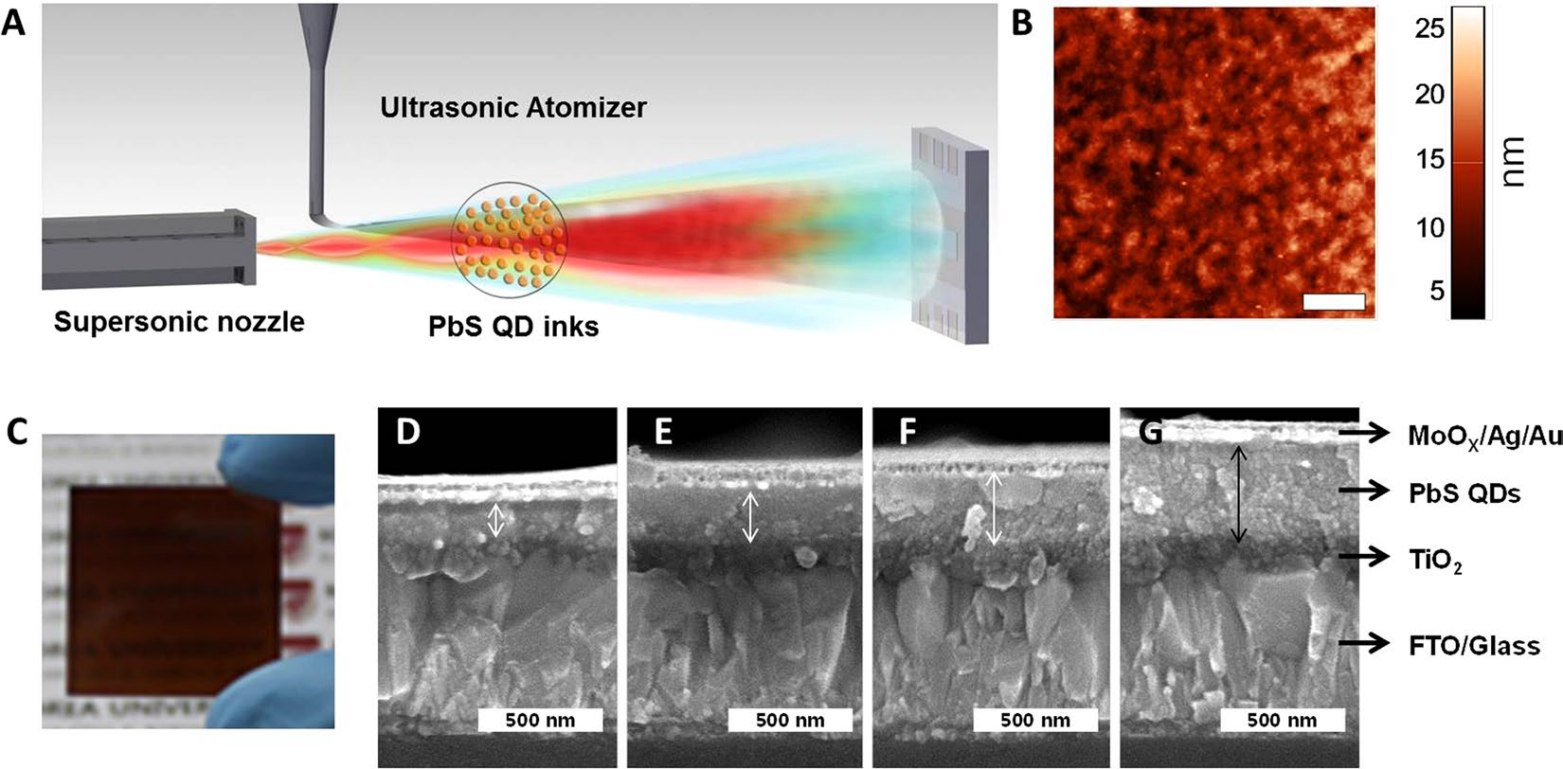
(**A**) Schematic of spray deposition of PbS QD ink. (**B**) AFM image (scale bar represents 1 µm), (**C**) photographic image, and (**D**–**G**) cross-sectional SEM images (scale bars represent 500 nm) of PbS QD films deposited by spray coating for (**D**) 8 sweeps, (**E**) 10 sweeps, (**F**) 12 sweeps, and (**G**) 14 sweeps. Average film roughness is 3.4 nm.

PbS QD inks can be used directly to rapidly form simple conductive films without further ligand exchange, whereas the PbS QDs-oleate system requires an LBL process for ligand exchange, as depicted in Fig. S8. Herein, the PbS QD ink films were fabricated *via* a spray-coating method under ambient conditions, as shown in Fig. 3A and Video SI. During the supersonic spraying process, heat and pressure are converted into kinetic and adhesion energies^26–28^. The spraying process allows for the direct deposition of the PbS QDs without requiring the use of any binder material. More importantly, during the spraying process, the air from the supersonic nozzle is heated to temperatures of up to 250 °C, which results in the rapid evaporation of the solvent and facilitates the deposition of the PbS QD ink, making a post-deposition annealing treatment for solvent removal unnecessary. Subsequently, the liquid droplets disintegrate quickly into finer ones because of the drag induced by the supersonic air stream. Thus, only the dried and crystallized QD particles hit the substrate at supersonic speeds, resulting in superior adhesion.

In order to obtain films of higher quality, we adjusted various parameters such as the nozzle temperature, solution concentration, and gas pressure. To begin with, by varying the nozzle temperature for PbS QD ink film deposition from 150 °C to 400 °C, we found that the optimal nozzle temperature is 250 °C, as shown in Fig. S9. Propylene carbonate, a solvent for QD inks, has a very high boiling point (242 °C). Thus, when the films were coated at temperatures lower than 250 °C, deposition did not occur even when the number of spraying passes was increased. Further, the films formed at temperatures of 300 °C or higher were not transparent enough even when the number of spraying passes was reduced and the duration of the passes kept short, in order to ensure that the films exhibited the desired thickness. Next, we fabricated PbS QD films at 250 °C while varying the PbS QD concentration to 3 and 10 mg mL^−1^. The concentration of the PbS QDs must be low enough to allow for the rapid evaporation of the carrier solution while ensuring the optimal dispersion of the residual PbS QDs. If the PbS QD concentration is too high, agglomeration may occur. Lastly, we fabricated PbS QD films at 250 °C while varying the gas pressure to 3 and 4 bar. The change in the operating pressure resulted in changes in the velocity of the jet stream. At higher pressures, the jet stream was faster; thus, the impact of the droplets of the PbS QD ink against the substrate was stronger, resulting in the formation of denser films.

Figure 3B shows atomic force microscopy (AFM) images of the close-packed PbS QD films with a thickness of 300 nm deposited on glass substrates by spray coating. It can be seen that the PbS QD films have a smooth topography with a root-mean-square (RMS) roughness of 3.4 nm; this is indicative of the high quality of the spray-deposited films. In addition, the film thickness could be controlled readily based on the number of passes/sweeps made during the spraying process; films with thicknesses of 130–400 nm could be obtained with 8–14 sweeps (Fig. 3D–G). A PbS QD ink solution with a concentration of only 3 mg mL^−1^ was supplied to the atomizer at a flow rate of 2 mL·min^−1^, and the spraying time required per pass was merely 4 s for an area with dimensions of 2.5 × 2.5 cm^2^ (see Experimental Section). The number of spraying passes/sweeps, *N*, required to deposit a 300-nm-thick film was 12. Therefore, the total amount of PbS QD ink used was 5 mg, and the entire deposition process took less than 1 min. Thus, the proposed supersonic spraying process can produce uniform PbS QD films by rapid and simple spraying using only a small amount of QD ink, as compared to other LBL processes involving spin and spray coating, which typically require more than 30 min to produce QD films with similar thicknesses^4, 13, 14^. Further, we evaluated whether the proposed method was suitable for forming flexible, large-area films by depositing the PbS QD ink to form a film on a polyethylene terephthalate (PET) substrate (10 × 6 cm^2^). As can be seen from Fig. S10, the proposed method has distinct advantages over conventional spin-coating methods.

### Characterization of PbS QD films

**Figure 4:**
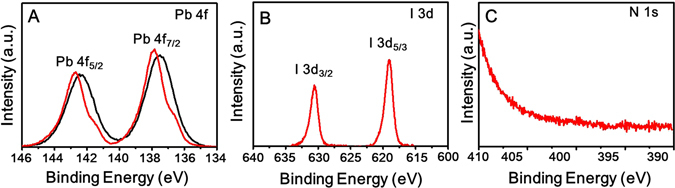
Binding energies of (**A**) Pb 4f, (**B**) I 3d, and (**C**) N 1 s as determined by XPS analysis of PbS QDs capped with oleate (black line) and MAPbI_3_ (red line) ligands.

The native oleate ligands were completely replaced by MAPbI_3_, as confirmed by Fourier transform infrared (FTIR) spectroscopy (Fig. S11). Prior to the ligand exchange, peaks related to several stretching and vibration modes of the oleate ligands were observed in the IR spectrum, such as the C–H and O–H stretching modes (2800–3500 cm^−1^), the C–H bending vibrations, and the carboxylic C–O and vinyl C = C stretching modes (600–1500 cm^−1^); however, all the peaks related to the stretching modes corresponding to the original ligands disappeared after the ligand exchange. The X-ray diffraction (XRD) pattern of the MAPbI_3_-stabilized PbS QD ink film was similar to that of the PbS QDs-oleate film. (Fig. S12). Notably, no peaks related to MAPbI_3_ perovskite solids and I-containing phases of other impurities were observed. X-ray photoelectron spectroscopy (XPS) was used to further confirm the chemical environment of the PbS QDs capped with MAPbI_3_ and compare it with that of the PbS QDs-oleate (Fig. 4A–C).

The binding energy of the Pb 4f_7/2_ species was higher by approximately 0.4 eV in the case of the PbS QDs-oleate as compared to that (137.9 eV) in the case of the MAPbI_3_-stabilized PbS QDs; this can be attributed to two factors: the fact that the PbI_3_-PbS QD films exhibited higher conductivity as compared to the insulating PbS QDs-oleate and to the formation of Pb-I bonds by the MAPbI_3_ ligands^29^. More importantly, a difference was observed in the shapes of the XPS spectra of the PbS QDs before and after the ligand-exchange reaction. We deconvoluted the measured XPS spectra of the PbS QDs-MAPbI_3_, as shown in Fig. S13, and found that the Pb 4f_7/2_ core level comprises two chemical states, which are observed at 137.9 and 136.8 eV, respectively. The main peak is attributable to the Pb component of the PbS-PbI_3_ structure; however, the smaller one is associated with the metallic Pb decomposed from the PbI_2_ precursor during the ligand-exchange process. Metallic Pb-related peaks are often seen in the case of organometallic perovskite structures^30^. The XPS spectra showed I-related peaks at 619.02 and 630.53 eV, which corresponded to I 3d_5/2_ and I 3d_3/2_, respectively; however, no nitrogen atoms were detected (Fig. 4C and D). When viewed in combination, the FTIR and XPS data verified that the volatile methyl ammonium cations were removed after film fabrication, thus implying that only the PbI_3_ ligands passivate the surfaces of the PbS QDs in the solid state^9^. QD ink films were also formed by other coating methods such as spin coating and drop casting (Fig. S14), in order to determine whether the ligand would change with the coating method; however, no nitrogen atoms were detected in the films produced using these methods either, as shown in Fig. S15.

**Figure 5:**
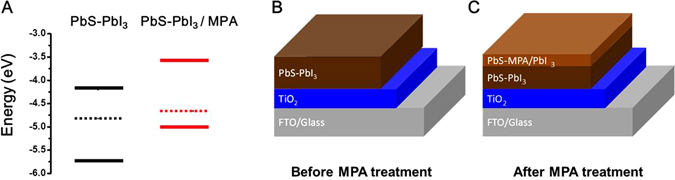
(**A**) Energy levels of conduction band minimum and valence band maximum and Fermi energy level versus vacuum level of PbS-PbI_3_ solids before and after MPA treatment, as determined by UPS measurements (Fig. S16). Structures of film of PbS QDs before (**B**) and after (**C**) MPA treatment.

Before fabricating photovoltaics using the PbS QD ink, ultraviolet photoelectron spectroscopy (UPS) was used to determine the electronic energy levels, such as the Fermi level energy (E_F_) and the valence band maximum (VBM), as functions of the vacuum level. Combining the UPS data and the optical bandgap data as determined from the absorption spectra (Fig. 2A) allowed us to determine the conduction band minimum (CBM). For the PbI_3_-stabilized PbS QD ink solids, E_F_ was located at 4.82 eV, while the energies corresponding to the CBM and VBM were 4.02 and 5.75 eV, respectively (Fig. 5A, black). Thus, based on the binding energy spectra, it can be surmised that the QD films formed were n-type films. Furthermore, we fabricated FETs using the PbI_3_-stabilized PbS QD ink solids to confirm the doping polarity. Source and drain Au contacts deposited on highly doped SiO_2_-coated silicon wafers were used as the substrates, and solutions of the QDs in PC were spray-coated at a nozzle temperature of 250 °C for 3 spraying passes to form ~50-nm-thick films. Figure S17 shows the output and transfer characteristics of a thus-fabricated FET, which clearly exhibits n-type behavior and linear/saturation curves. The electron mobility, *μ*_lin_, was determined to be approximately 8.3 cm^−2^ V^−1^ S^−1^ in the linear regime at V_DS_ = 2.5 V.

Based on previous studies, the I ions in iodide-capped Pb chalcogenide QD solids can be considered as n-type doping agents^9, 10^. Unfortunately, such PbS QD inks with n-type polarity cannot be used for fabricating p-n junction photovoltaics, wherein TiO_2_ and PbS QD films are typically used as the n-layer and p-layer, respectively. Thus, we treated the QD films with 3- mercaptopropionic acid (MPA), in order to change their doping polarity, resulting in p-type QD films, as indicated by the red lines in Fig. 5A (the energy levels corresponding to the E_F_, CBM, and VBM were 4.68, 3.57, and 5.02 eV, respectively). UPS is a highly surface-sensitive technique, wherein the escape depth is typically only a few angstroms; hence, only the surface layers are probed by this technique^31^. A small amount of the MPA solution (0.11 M, 0.25 mL) that was placed on the PbI_3_-capped QD films did fully infiltrate to the bottom of the QD films with a thickness of 300 nm. This suggested that the MPA-treated PbI_3_-capped QD films consisted of n-type PbS QDs in the bottom layers and p-type PbS QDs in the top layer, resulting in a graded architecture (n^+^-n-p), as shown in Fig. 5C.

Based on these results, the PbS QDs-MAPbI_3_ ink was deposited onto a ~80-nm-thick TiO_2_ layer as the n-layer *via* the spraying technique (Fig. 3A); the TiO_2_ layer was formed on a fluorine-doped tin oxide (FTO) glass. The coated substrate was subjected to a subsequent treatment with MPA in methanol; this was followed by the deposition of a MoO_3_/Au/Ag top contact. We evaluated the thickness dependence of the absorbing PbS layer by producing devices using different numbers of spraying sweeps, as mentioned above (Fig. 3C–F). After the spray-coating process, each film was treated with MPA. Thus, only the thickness of the PbI_3_-capped QD layer was changed. The open-circuit voltage (*V*_OC_), short-circuit current (*J*_SC_), fill factor (*FF*), and PCE are plotted as functions of the number of spraying sweeps in Fig. S18. The results indicate that the optimal thickness of the PbS QD ink film was approximately 300 nm, which was obtained after 12 sweeps. In particular, the *FF* value decreased after 10 sweeps, while the other parameters (*J*_*SC*_, *V*_*OC*_, and PCE) increased with the number of spraying sweeps, with their values being the highest in the case of the film with a thickness of 300 nm and subsequently decreasing for thicker films. This trend was in keeping with previous reports^8^. The dependence of the device performance on the film thickness is attributable to the high trap state density of the films, which results in a tradeoff between charge generation and collection.

**Figure 6:**
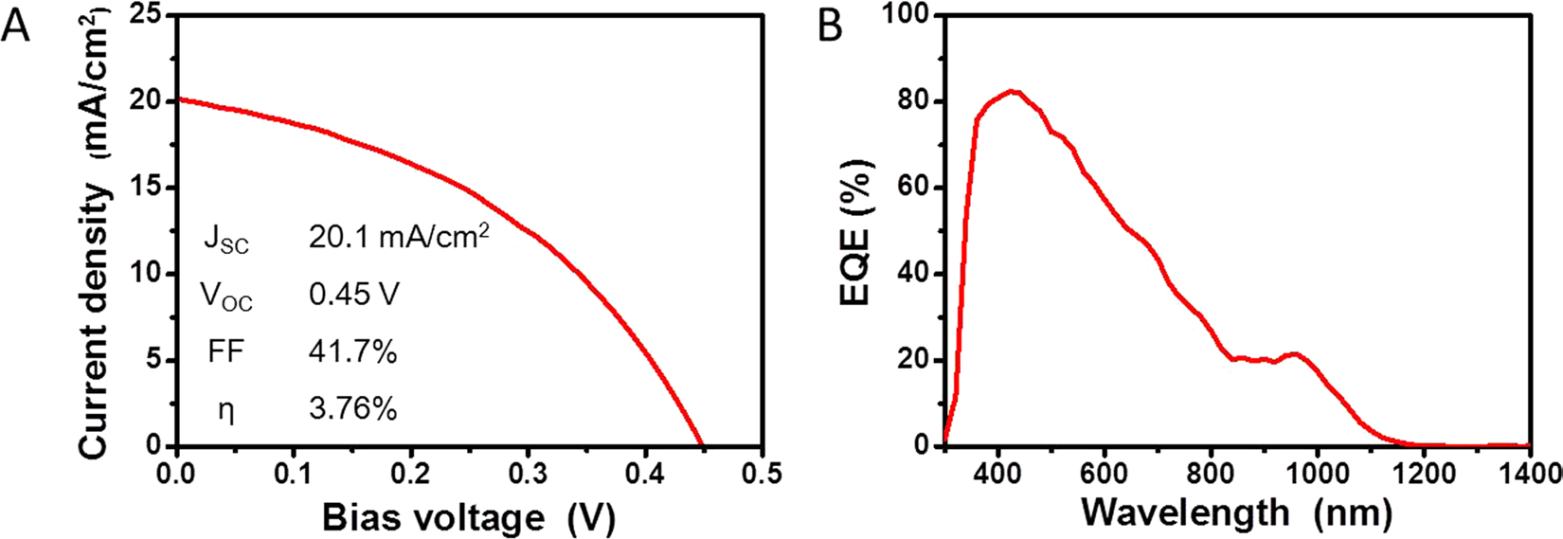
(**A**) *J*–*V* curves obtained under AM1.5G illumination and (**B**) EQE spectra of solar cells with PbS QD heterojunction with graded architecture and having the structure glass/FTO/TiO_2_/PbS-PbI_3__MPA/PbS-MPA/MoO_x_/Au/Ag, which was formed by spray deposition.

Figure 6 shows the *J–V* curves (a) and external quantum efficiency (EQE) spectra (b) of the optimized devices based on PbS QD ink films produced using MPA solutions of different concentrations and by rinsing in methanol (Fig. S19). The *J*_*SC*_, *V*_*OC*_, and *FF* values of the device were 20.1 mA·cm^−2^, 0.45 V, and 41.7%, respectively, with the PCE value (*η*) being 3.76%. The *J*_*SC*_ value under AM1.5 G conditions is similar to that (20 mA·cm^−2^) determined from the EQE spectrum; this also confirms that quantum confinement was maintained in the QD solids (Fig. 6B). Finally, a histogram of the performance distribution of the fabricated devices is shown in Fig. S20.

In conclusion, a high-quality PbS QD ink was synthesized successfully *via* the solution-phase ligand-exchange process while employing MAPbI_3_ as the precursor. In the colloidal form, the PbS QD ink is stabilized by both the PbI_3_ anions and the MA cations, with an electrical double layer being formed, resulting in the colloids remaining stable for months. On the other hand, the film fabrication process removes the volatile MA cations. Supersonic spray coating using a QD ink is thus an economically viable approach for the commercial manufacturing of solution-processed PbS QD solar cells while allowing for efficient thickness control and ensuring minimal PbS precursor consumption. In the PbS QD solids, the PbI_3_ ligands act as surface ligands as well as dopants, resulting in n-type behavior; treatment with MPA leads to a change in the doping polarity of the upper region of the films to p-type, leading to the formation of a structure with a graded architecture. The solar cells fabricated using the PbS QD ink exhibited a PCE value of 3.7%.

## Methods

### Materials

Lead (II) oxide (Aldrich, 99.999%), oleic acid (OA, Alfa Aesar, 99%), 1-octadecene (ODE, Aldrich, 90%), TMS_2_S (Aldrich, 99.999%), methylammonium iodide (MAI, Aldrich, 98%), lead iodide (Aldrich, 99%), NMF (Aldrich, 99%), PC (Sigma-Aldrich, 99.7%), tetrachloroethylene (TCE, Aldrich, ≥99%), titanium(IV) *N*-butoxide (Aldrich, 97%), triethanolamine (Sigma, ≥99.0%), zirconium ethoxide (Aldrich, 97%), and acetic acid (Sigma-Aldrich, ≥99.99%) were the materials used in this study.

### Synthesis of oleate-passivated PbS QDs

All the manipulations were performed using the standard Schlenk line techniques. In a typical synthesis process, lead oxide (0.46 g, 2 mmol), OA (1.5 mL, 4.5 mmol), and ODE (10 mL) were mixed in a three-neck flask, degassed under vacuum, and heated to 110 °C for 2 h. Thereafter, the flask was heated to 115 °C in a nitrogen atmosphere. Next, TMS_2_S (210 µL, 1 mmol) in 4 mL of ODE was loaded into a syringe in a nitrogen-filled glove box and rapidly injected into the solution at 110 °C after removing the mantle. Next, the flask was transferred to a glove box, and acetone was added to the reaction solution to isolate the PbS QDs by precipitation. The resulting precipitate was washed twice with acetone to remove the residual surfactant and reaction debris and was then dispersed in octane at a concentration of 100 mg·mL^−1^ for use in the solution-state ligand-exchange process.

### Ligand-exchange process (synthesis of PbS QD ink)

The ligand-exchange process was performed as per a previously reported procedure^16^. In a nitrogen atmosphere, a solution of 1.58 g (10 mmol) of MAI and 4.61 g (10 mmol) of PbI_2_ in 10 mL of NMF was stirred for 30 min at 80 °C until all the ingredients had dissolved completely. The ligand solution was then mixed with 5 mL of a hexane solution of the PbS QDs capped with oleate ligands in a vial. The biphasic solution was stirred for approximately 30 min at 100 °C until all the PbS QDs had migrated from the octane phase to the NMF phase. The nonpolar octane phase was removed and the polar PbS QDs in the NMF phase were rinsed thrice with pure hexane. To remove the excess PbMAI_3_ ligands, the PbS QDs were precipitated by adding acetone and then redispersed in PC.

### Supersonic spray deposition

Figure 3(A) illustrates the entrainment of the PbS precursor droplets from the ultrasonic atomizer and their subsequent injection into the supersonic gas stream emanating from the supersonic nozzle. Once the PbS liquid droplets were injected into the supersonic stream, the droplets underwent secondary fragmentation because of the severe drag experienced by them because of the high-speed gas. Owing to this secondary fragmentation, the solvent evaporated rapidly, and only the dried residuals were deposited onto the substrate. The supersonic stream was obtained by using a high chamber pressure (4 bar) and heating the gas to 250 °C; this energy was converted into kinetic energy by the converging/diverging supersonic nozzle. Details of the supersonic spray-deposition process are given in our previous publications^26–28^. The PbS QD precursor was supplied to the atomizer at a flow rate of 2 mL·min^−1^ and atomized. In the case of a single supersonic nozzle with a diameter of 2 mm, 10 spraying passes/sweeps were required to deposit a 200-nm-thick film with an area of 2.5 × 2.5 cm^2^. The duration of each spraying pass was 4 s; therefore, the total amount of solvent used to fabricate a single film with an area of 2.5 × 2.5 cm^2^ and thickness of 200 nm using the precursor solution with a concentration of 3 mg·mL^−1^ was 1.3 mL. The deposition is a series of successive passes and there are no pauses in between during the spray coating.

### Preparation of TiO_2_ films

Prior to the fabrication of the heterojunction solar cells, the FTO-coated glass substrates were cleaned in a sonication bath using a mixture of acetone, methanol, and deionized water. The Zr-doped TiO_2_ nanoparticles were synthesized using a previously documented non-hydrolytic sol-gel method^32^. The prepared Zr-doped TiO_2_ nanoparticles were spun onto the patterned FTO substrates by placing 20 drops of the Ti-sols and spin-coating at 2500 rpm for 15 s. The resulting films were annealed at 450 °C for 30 min.

### Fabrication of PbS QD ink solar cells

The PbS QD ink was deposited onto the TiO_2_/FTO/glass substrates *via* supersonic spray coating, as mentioned above. Next, 5 drops of a solution of MPA in methanol in a concentration of 1 vol% were placed onto the PbS QD ink films and spin-coated at 2500 rpm for 10 s. This was followed by the spin-coating of 10 drops of pure methanol at 2500 rpm for 30 s. Subsequently, MoO_3_ (15 nm), Au (50 nm), and Ag (100 nm) electrodes were formed by thermal evaporation (<10^−7^ Torr) on the MPA-treated QD films using a shadow mask.

### Optical characterization of QDs

The absorption measurements were performed using a Thermo Scientific EVOLUTION 201 spectrophotometer. The PL data were collected using a high-power Xe light source (HPX-2000, optical power >150 mW for visible range, 785 nm spectrum stabilized, laser module power >350 mW for IR range) and detector (Maya 2000 Pro for visible range, NIR Quest for IR range). The DLS measurements for particle size analysis were performed with a NANOPHOX (NX0046) spectrometer.

### Structural characterization of QDs (TEM and XRD analyses)

Electron micrographs of the QDs were obtained on a carbon-coated Cu mesh grid using a Tecnai F30 Super-Twin system (FEI Co., Hillsboro, OR, USA; Yun-Chang Park, KAIST NanoFab). The XRD patterns were obtained using a Rigaku Ultima III diffractometer equipped with a rotating anode and a Cu-Kα radiation source (λ = 0.15418 nm).

### Photoemission spectroscopy measurements (XPS and UPS analyses)

The base pressure of the analysis chamber for the spectroscopic measurements was kept at less than 10^−10^ Torr. The UPS and XPS measurements were performed using a hemispherical electron energy analyzer with a charge-coupled device camera (SES-100, VG-Scineta). The UPS measurements used a HeI (hω = 21.22 eV) gas discharge lamp as the excitation source, with the sample bias for the secondary electron cut-off region being −10 V. The XPS measurements used an Al-Kα (hω = 1486.5 eV) radiation source without a monochromator. The energy resolutions were 0.1 and 1.0 eV, respectively.

### Device characterization

The current-voltage curves of the fabricated devices were recorded using a Keithley 2400 source meter. A solar simulator (94023 A, New port INC) with a 450 W Xe lamp as the light source and operating at 100 mW·cm^−2^, as calibrated using a standard silicon solar cell, was employed to simulate the solar spectrum under AM1.5G conditions. The pre-sweep delay time was 1 s and the dwell time for each voltage step was 200 ms. A total of 40 data points were measured between −0.1 and 0.70 V in both the forward and the reverse direction. The EQE measurements were performed using a custom-built set-up (SPIQE200-5327, Newport INC) comprising a 300 W Xe lamp, a power supply, a monochromator with a bandwidth of 20 nm, and a Merlin lock-in amplifier. A beam with an area of 0.01 cm^2^ was used to excite the solar devices with an active area of 0.03 cm^2^. All the current-voltage and EQE measurements were performed in a glove box filled with nitrogen.

## Electronic supplementary material

Fig S1, Fig S2, Fig S3, Fig S4, Fig S5, Fig S8, Fig S7, Fig S8, Fig S9, Fig S10, Fig S11, Fig S12, Fig S13, Fig S14, Fig S15, Fig S16, Fig S17, Fig S18, Fig S19, Fig S20
