# Supplementary material for: Supersonically Spray-Coated Colloidal Quantum Dot Ink Solar Cells
Source: Sci Rep. 2017 Apr 4;7:622. doi: 10.1038/s41598-017-00669-9 (PMC5428848; doi:10.1038/s41598-017-00669-9)
Supplement: Supplementary file 1 — Fig S1, Fig S2, Fig S3, Fig S4, Fig S5, Fig S8, Fig S7, Fig S8, Fig S9, Fig S10, Fig S11, Fig S12, Fig S13, Fig S14, Fig S15, Fig S16, Fig S17, Fig S18, Fig S19, Fig S20 [file 41598_2017_669_MOESM1_ESM.pdf]

Supporting Information

Supersonically Spray-Coated Colloidal Quantum Dot Ink Solar Cells

Hyekyoung Choi [1,2], Jong-Gun Lee[3], Xuan Dung Mai[1,4], Matthew C. Beard,[5] Sam S. Yoon[3*], and Sohee Jeong[1.2*]

[1]Nano-Mechanical Systems Research Division, Korea institute of Machinery and Materials (KIMM), Daejeon, 305-343, Republic of Korea.

[2]Korea University of Science and Technology (UST), Daejeon 305-350, Republic of Korea.

[3]School of Mechanical Engineering, Korea University, Seoul 136-713, Republic of Korea.

[4]Department of Chemistry, Hanoi pedagogical university No 2, Vinh Phuc, Vietnam.

[5]National Renewable Energy Laboratory, Golden, Colorado 80401, United States

*skyoon@korea.ac.kr & sjeong@kimm.re.kr

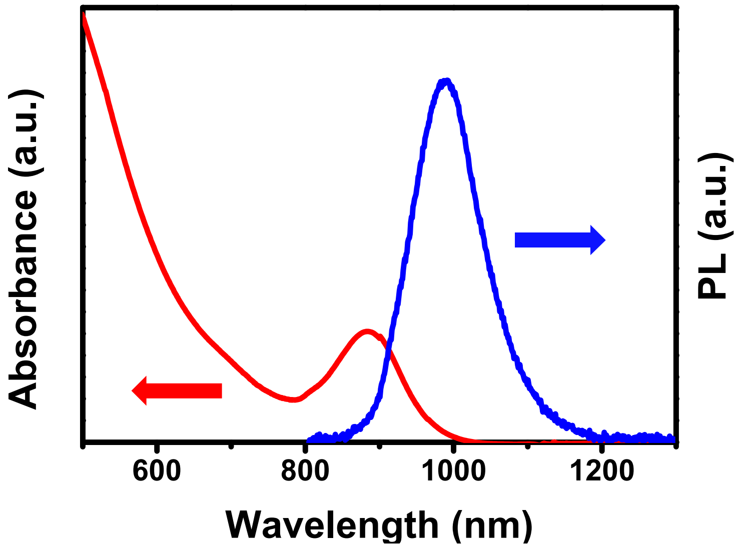

Figure S1. Absorbance (red line) and PL (blue) spectra of pristine PbS QDs capped with oleate dispersed in tetrachloroethylene.

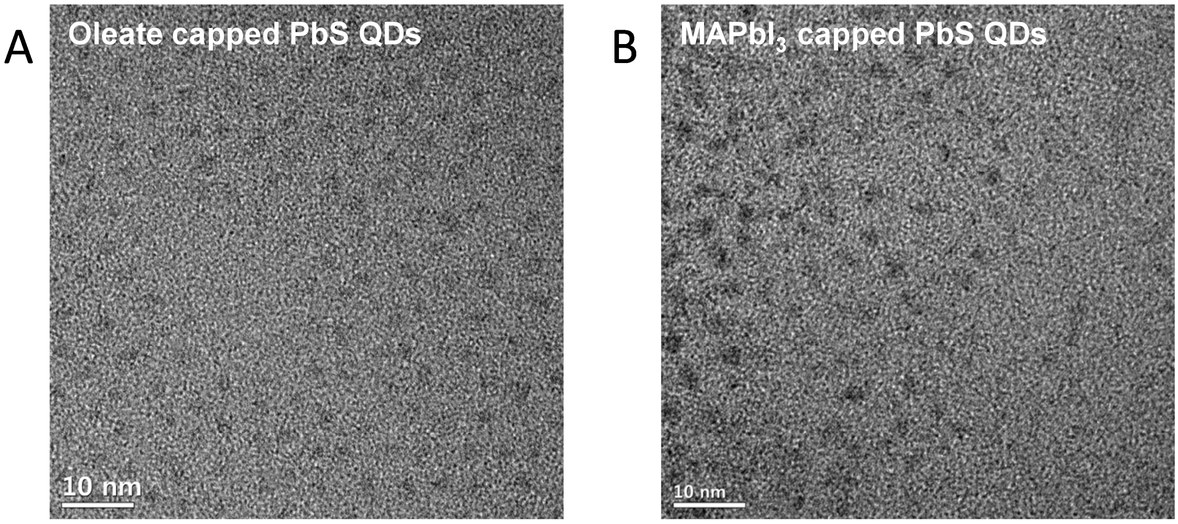

Figure S2. TEM images of PbS QDs capped with oleate (A) and $MAPbI_3$ (b) (Scale bars = 10 nm). The oleate and $MAPbI_3$ capped PbS QDs have sizes of 3.4 ± 0.25 nm and 3.4 ± 0.28, respectively.

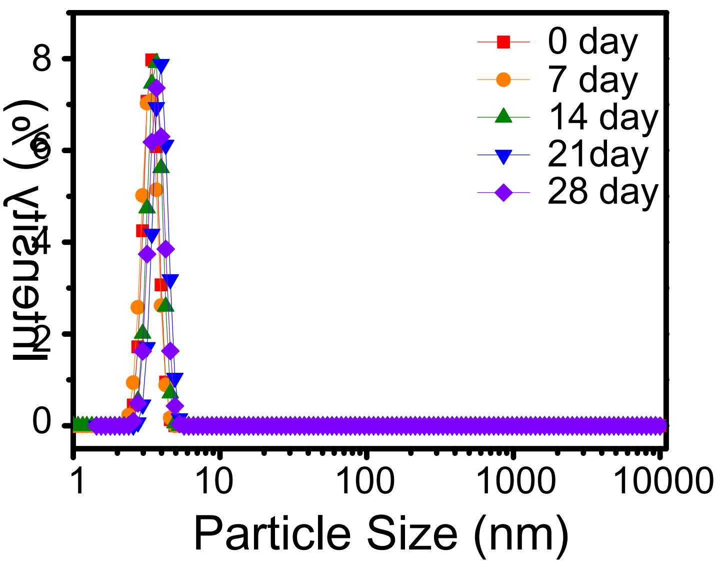

Figure S3. Hydrodynamic particle sizes of PbS QD inks-$MAPbI_3$ monitored by dynamic light scattering (DLS) measurement after ligand exchange (red), and after 7 (orange), 14 (green), 21 (blue), and 28 (purple) days.

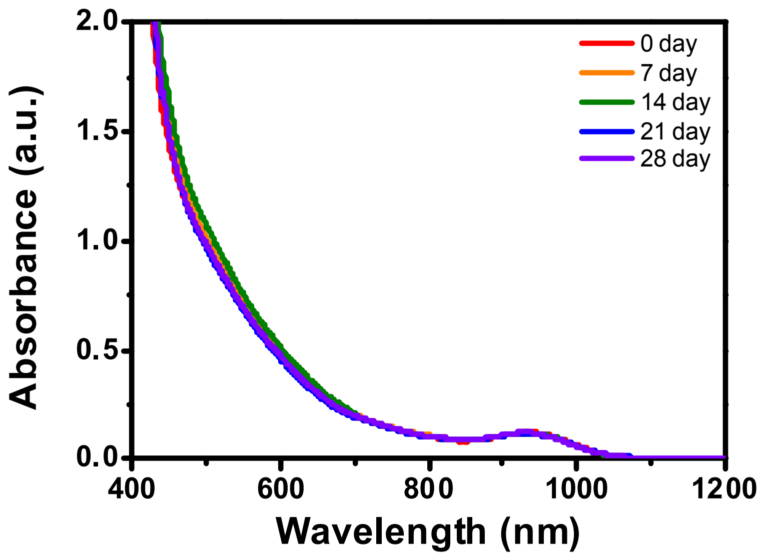

Figure S4. Absorption spectra of PbS QD inks-$MAPbI_3$ dispersed in propylene carbonate (PC), monitored after ligand exchange (red), and after 7 (orange), 14 (green), 21 (blue), and 28 (purple) days.

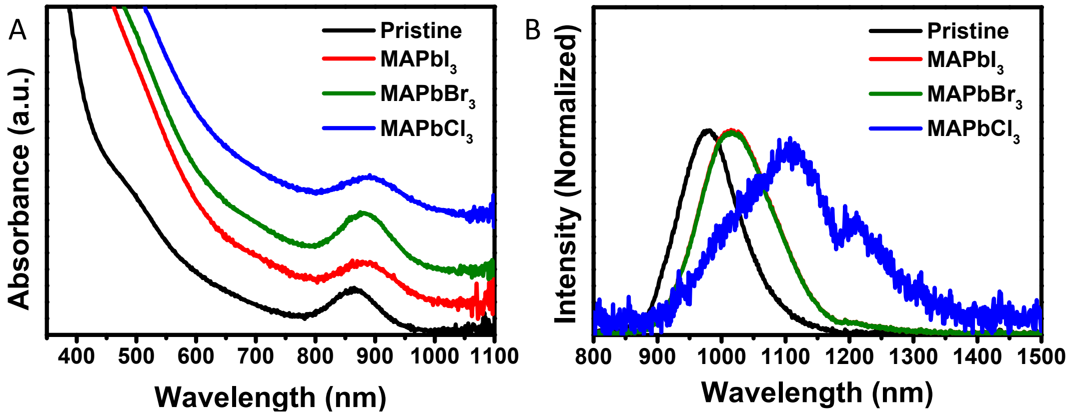

Figure S5. Absorbance (a) and PL (b) spectra of PbS QDs capped with oleate (black), $MAPbI_3$ (red), $MAPbBr_3$ (green), and $MAPbCl_3$ (blue).

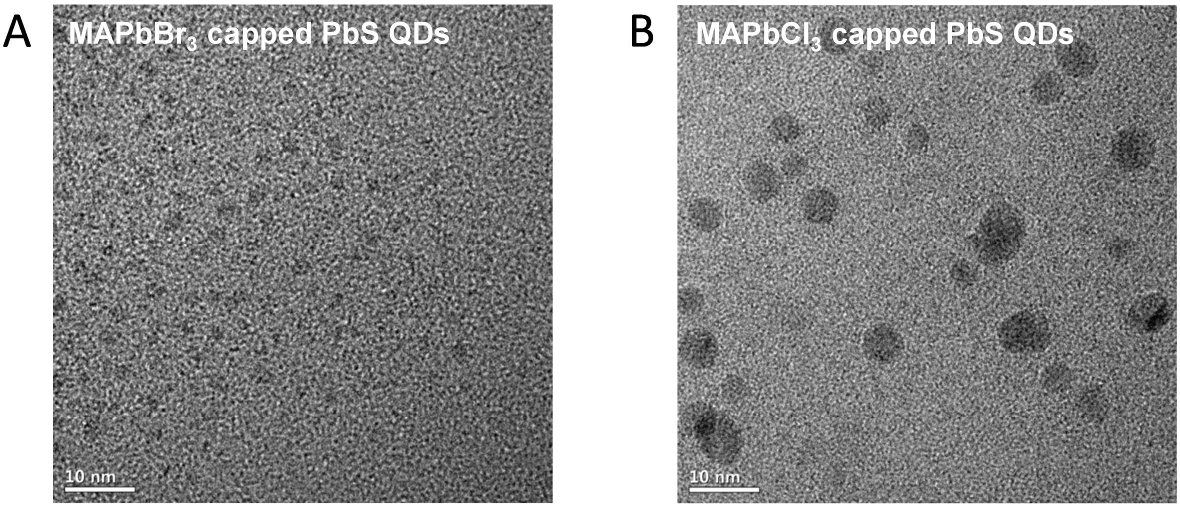

Figure S6. TEM images of PbS QDs capped with $MAPbBr_3$ (A) and $MAPbCl_3$ (b) (Scale bars = 10 nm). The $MAPbBr_3$ and $MAPbCl_3$ capped PbS QDs have sizes of 3.4 ± 0.28 nm and 4.2 ± 1.23, respectively.

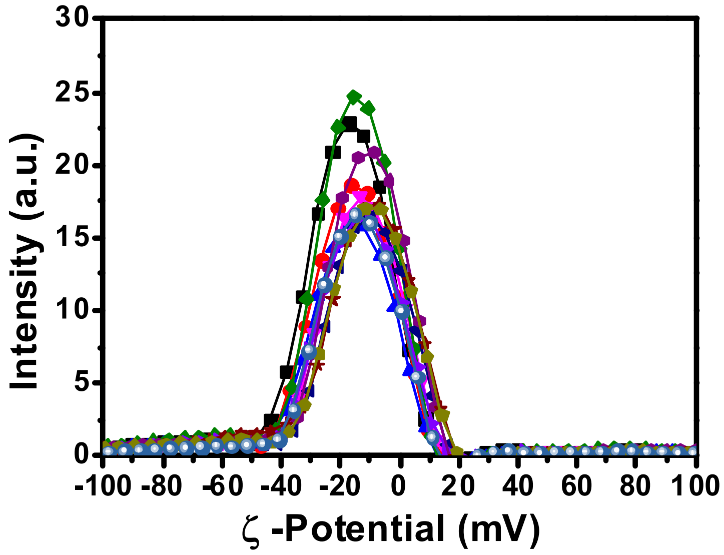

Figure S7. ζ-potential distribution of PbS QDs capped with $MAPbI_3$ in propylene carbonate, showing averaged -13 mV from 11 samples.

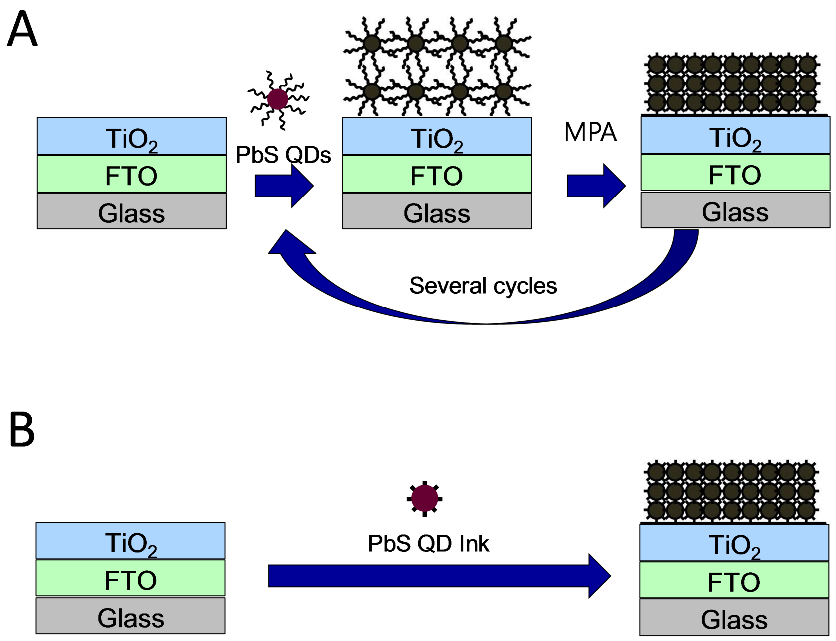

Figure S8. Schematic of the standard layer-by-layer process with pristine PbS QDs-oleate (a) and directly deposition process with PbS QD inks-$MAPbI_3$ on the substrates (TiO_2/FTO/Glass) for heterojunction photovoltaics.

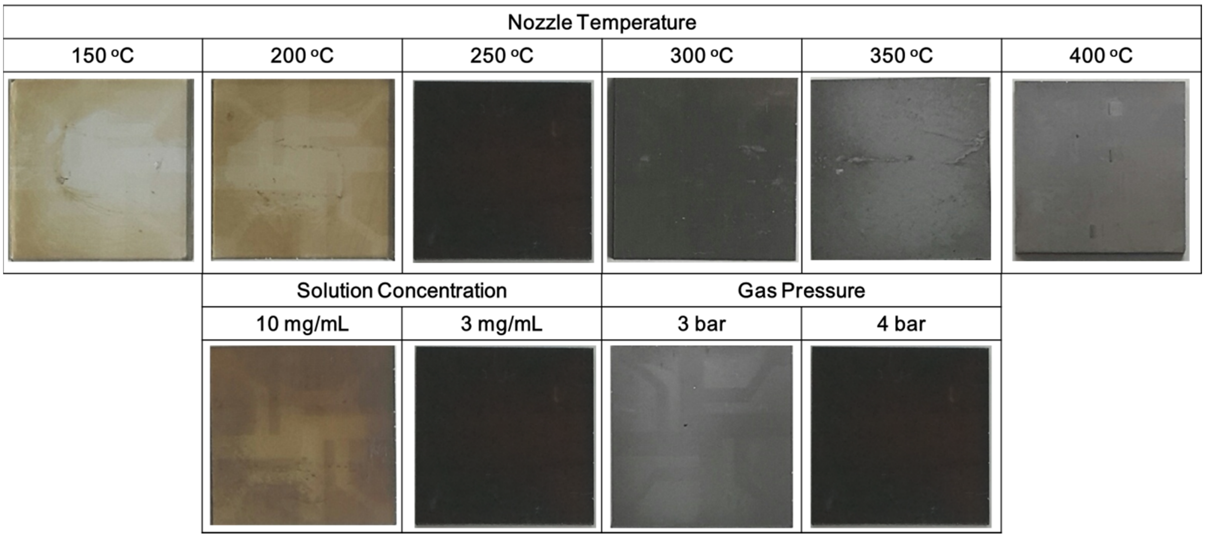

Figure S9. Photos of PbS QD films deposited on the FTO/glasses depending on nozzle temperature (150 – 200 ^{o}C), solution concentration (10, 1 mg mL^{-1}), and gas pressure (3, 4 bar) for spray coating.

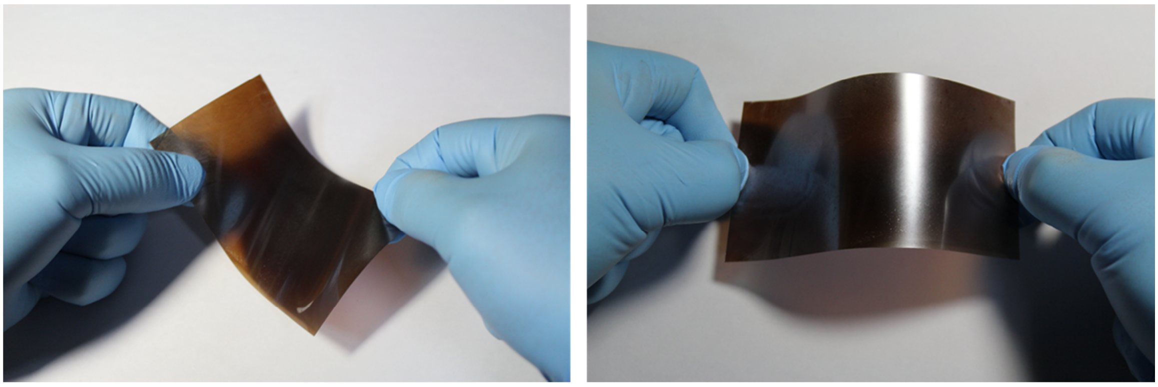

Figure S10. Photos of PbS QD films deposited on the PET substrate.

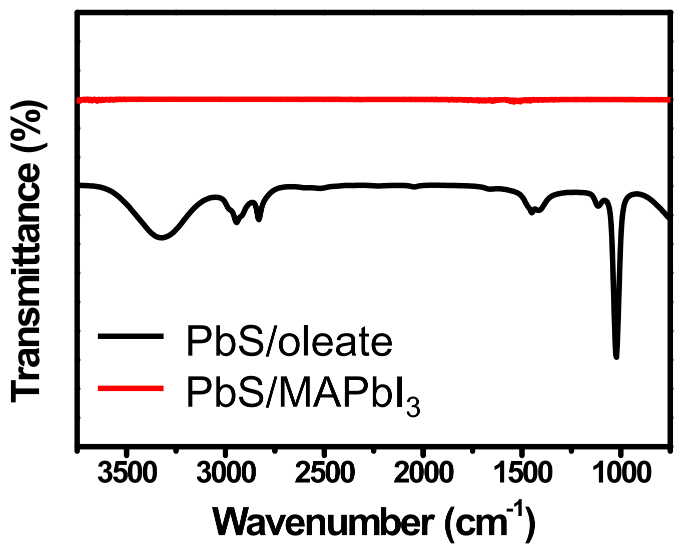

Figure S11. FT-IR spectra of PbS QDs capped with oleate (black) and $MAPbI_3$ (red).

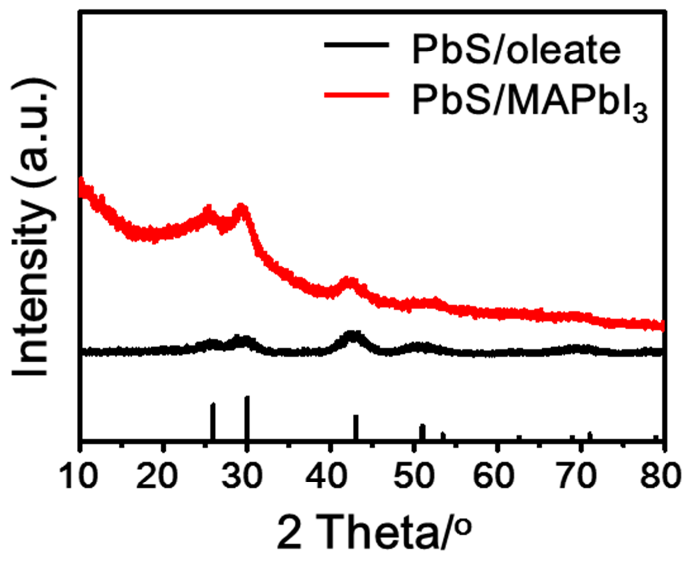

Figure S12. (a) XRD patterns of PbS QDs capped with oleate (black line) and capped with MAPbI3 (red line). Bars at the bottom indicate peaks of bulk rock salt structure of PbS.

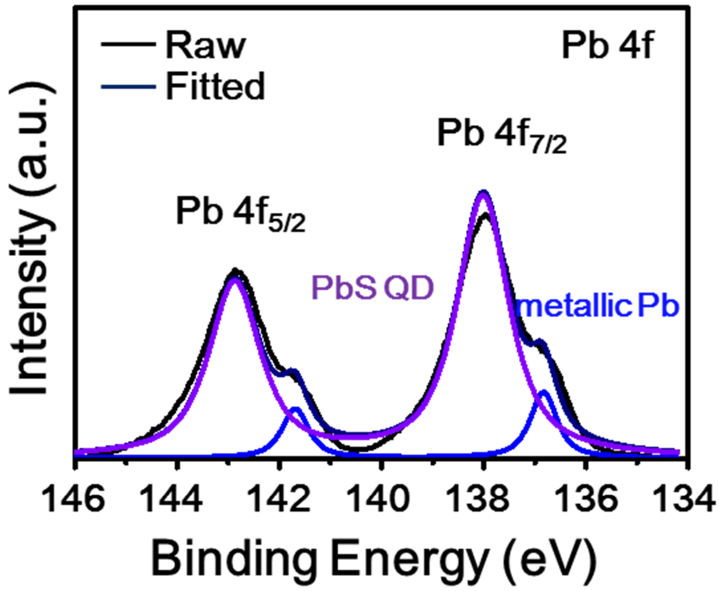

Figure S13. Raw and fitted curves of high-resolution Pb 4f XPS spectra for PbS QDs capped with PbI_3 after ligand exchange reaction.

.

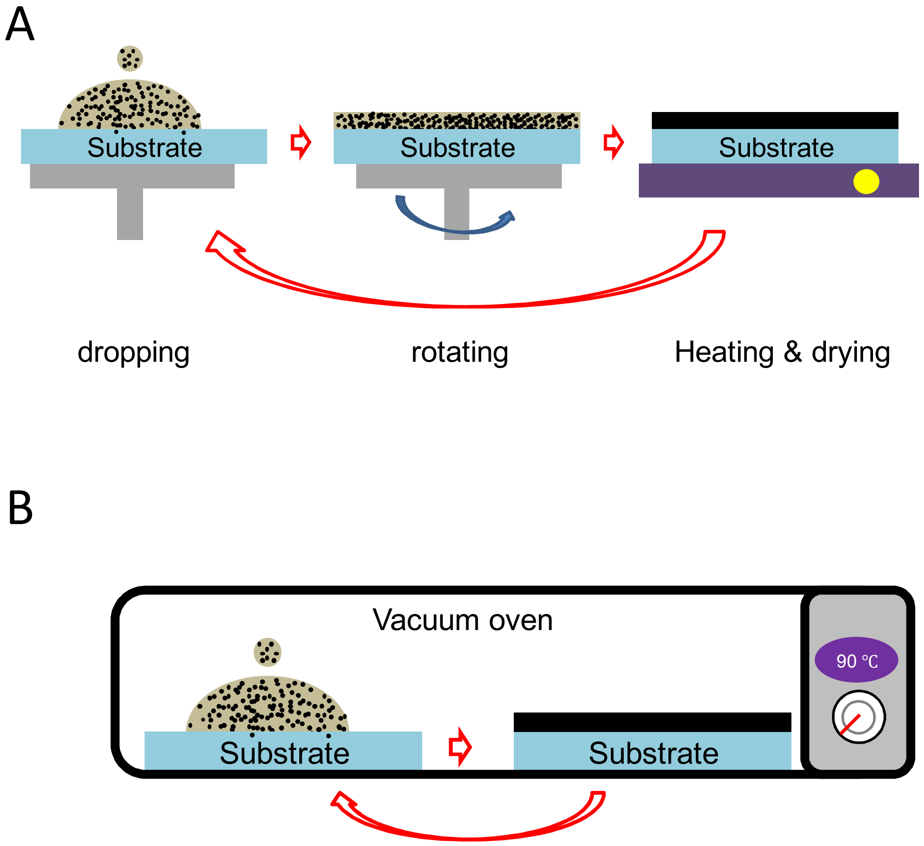

Figure S14. Schematic of film fabrication *via* the spin coating method (a) and drop casting method (b) with PbS QD inks- $MAPbI_3$.

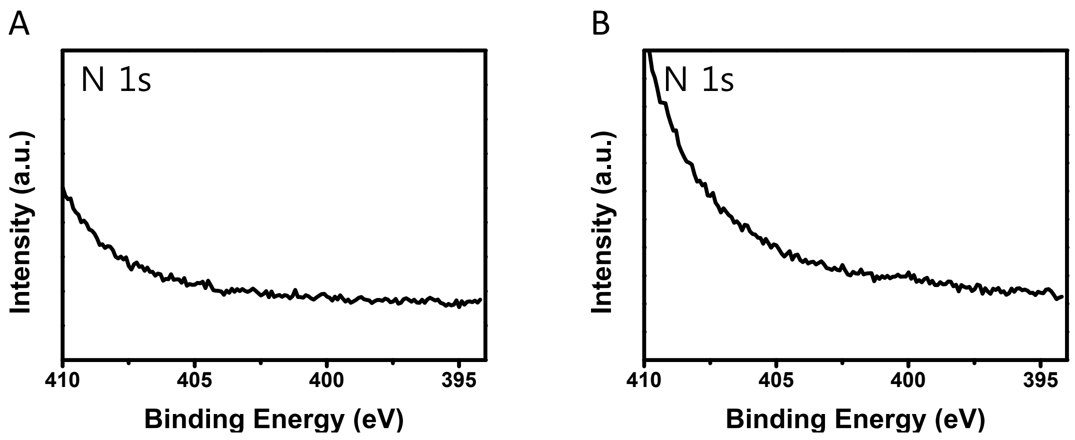

Figure S15. Binding energy spectra of N 1S from XPS measurement of PbS QD ink films deposited by spin coating method (a) and drop casting method (b).

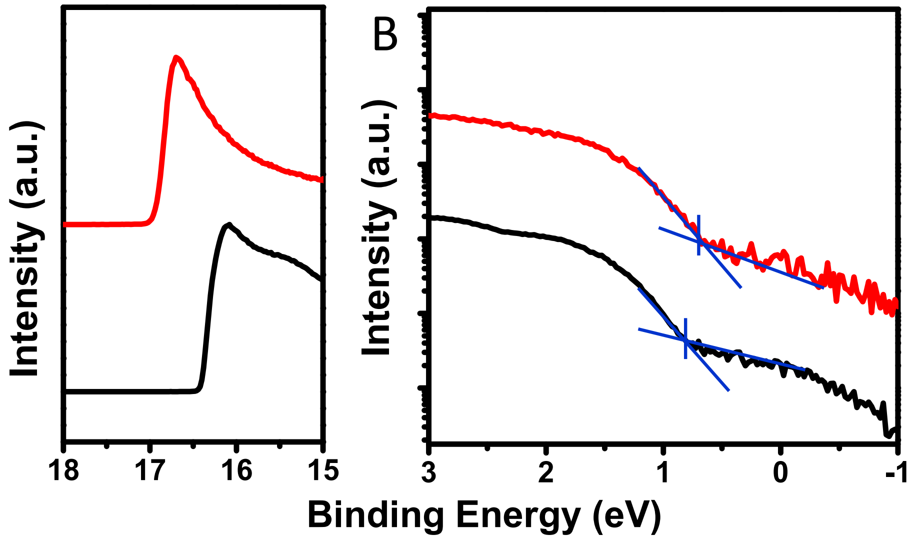

Figure S16. Binding energy spectra from UPS measurement. (a) Secondary cut-off and (b) valence band region of PbS QD ink solids before (black) and after (red) MPA treatment process.

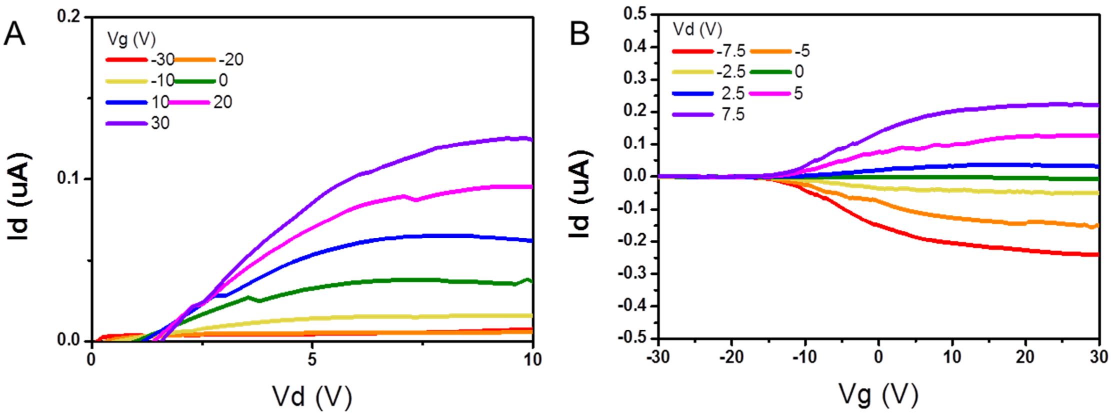

Figure S17. Representative output (a) and transfer (b) characteristics of the n-channel FET using PbI_3 capped PbS QD films without any treatment.

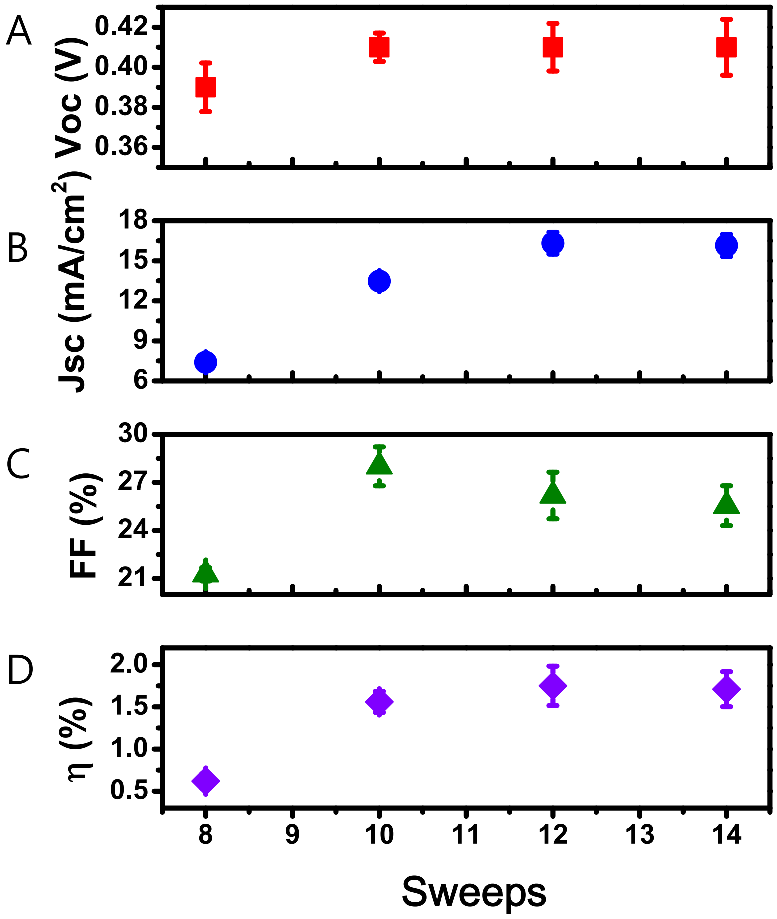

Figure S18. Variation of key solar cell parameters of (a) open-circuit voltage (V_{OC}), (b) current density (J_{SC}), (c) fill factor (FF), and (d) power conversion efficiency (PCE) with PbS QD ink solar cells dependent on the number of sweeps (8 – 14) in spray coating.

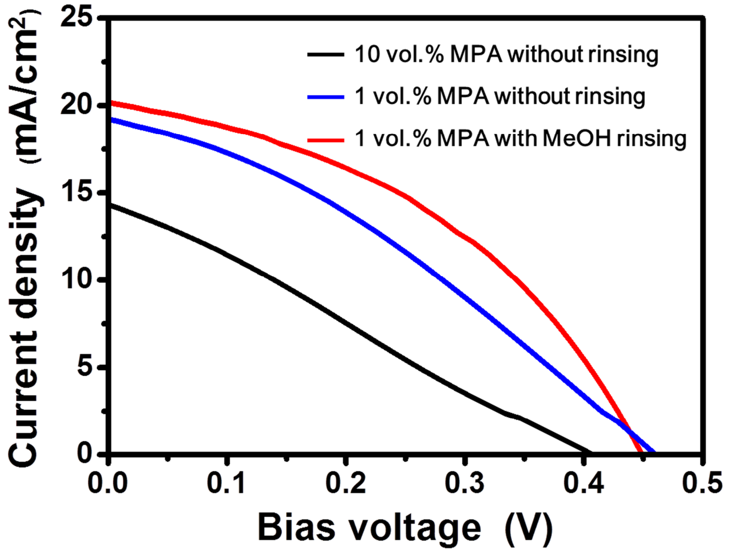

Figure S19. J-V curves of the solar cells using 10 vol.% MPA solution without rinsing (black), 1vol.% MPA solution without rinsing (blue), and 1 vol.% MPA solution with pure MeOH rinsing (red).

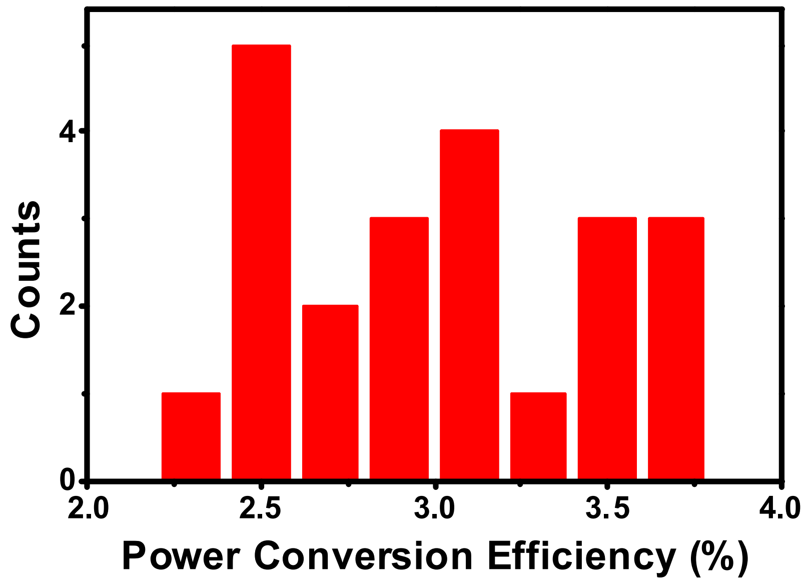

Figure S20. Histogram of power conversion efficiencies for 22 separate devices under AM 1.5G illumination conditions of the PbS QD ink hetero-junction solar cells.
